# Clinical and radiological responses to oral methotrexate alone or in combination with other agents in Erdheim-Chester disease

**DOI:** 10.1038/s41408-017-0034-7

**Published:** 2017-12-15

**Authors:** Gaurav Goyal, Mithun V. Shah, Timothy G. Call, C. Christopher Hook, William J. Hogan, Ronald S. Go

**Affiliations:** Division of Hematology, Mayo Clinic, Rochester, MN USA

Erdheim-Chester disease (ECD) is a rare histiocytic disorder that is now included under hematopoietic neoplasm according to 2016 World Health Organization classification^1^. Recently, identification of targetable MAP kinase pathway mutations like *BRAF-V600E* in ECD has changed the initial treatment approach in 50–70% of patients^2, 3^. MEK inhibitors seem promising in patients with other activating MEK-ERK pathway mutations, but with limited experience^4, 5^. However, there is a paucity of data on therapeutic options in patients who do not harbor these mutations, who relapse after targeted therapy, and who do not have adequate tissue available for genomic testing. Historically, interferon alfa and cladribine have been utilized with variable efficacy^6, 7^. There have been some reports of successful treatment of Langerhans Cell Histiocytosis with oral methotrexate^8, 9^. In this study, we report our institution’s experience with oral methotrexate in treatment of ECD.

After obtaining consent waiver and approval from the institutional review board, we retrospectively reviewed the medical records of ECD patients evaluated at Mayo Clinic from 1 January 1998 to 6 April 2016 who received methotrexate. The diagnosis of ECD was made by using clinical criteria in conjunction with histopathologic findings^10^. All the biopsy specimens were independently reviewed at our institution.

Since post-therapy response assessment was not uniformly performed, we used previously published clinical and radiological response criteria^7^. The clinical responses were divided into: (i) complete freedom from symptoms attributed to ECD (complete response or CR); (ii) significant improvement, but not complete resolution, of the symptoms attributed to ECD (partial response or PR); (iii) no significant change in the symptoms attributed to ECD (stable disease or SD); (iv) significant worsening of symptoms attributed to ECD (progressive disease or PD). The minimum duration for symptom improvement to be considered a response was arbitrarily set at 3 months.

Radiological response to therapy was assessed using the imaging studies available. In our series, a large proportion of patients did not undergo positron emission tomography–computed tomography (PET-CT) scans and responses were assessed based on alternative imaging studies available: X-rays, magnetic resonance imaging, computed tomography scans, and radionuclide bone scans. The radiologic responses were categorized as follows based on the change in the size or appearance of lesion(s) suspected/proven to be ECD: (i) CR: complete resolution; (ii) PR: significant improvement (decrease in activity or size by any degree), but not complete resolution; (iii) SD: no significant change for ≥3months); and (iv) PD: significant progression.

A total of 63 patients with ECD were identified. The median age at diagnosis was 54 years (range, 34–80 years). Methotrexate was administered to 13 (21%) of the patients. The median age at diagnosis of the latter cohort was 51 years (age range, 34–66 years), and 7 (54%) were male. Organs involved included: bone (85%), central nervous system (54%), kidney/retroperitoneum (54%), cardiovascular system (38%), lung (23%), and skin (23%; Table 1). Methotrexate was administered as first-line therapy for six patients and second/subsequent line of therapy for the remaining seven patients (range, 1–6). The median duration of therapy was 4 months (range, 1–163). The most commonly used dose for methotrexate was 15 mg/week orally (range, 7.5–25 mg). It was administered as a single agent in three patients, in combination with prednisone in six patients, and in combination with other biologics (etanercept or infliximab) in four patients.

**Table 1:** Individual patient characteristics for 13 Erdheim-Chester disease patients treated with methotrexate (MTX)

	Age at diagnosis/sex	Sites/organs involved	Prior therapies	Treatment	Weekly oral dose of MTX	Duration of MTX (months)	Clinical response	Radiologic response	*BRAF V600E* mutation	Comments	Dead/alive (age)
1	51/M	Pharynx, bones/joints	None	MTX + prednisone + etanercept/infliximab	17.5 mg	15	PD	PD	ND	Increase in size of the pharyngeal lesion, arthralgia	Alive (58)
2	64/F	Bone, bone marrow, lung, liver, spleen, lymph nodes, CVS	None	MTX + Infliximab	20 mg	4	PD	PD	Present (PCR)	Worsening symptoms, splenomegaly and lymphadenopathy	Alive (67)
3	39/M	Orbit	None	MTX + prednisone	25 mg	3	PD	PD	ND	Worsening eye symptoms and PET-CT	Dead (41)
4	47/F	Bone, CNS, lung, kidney, retroperitoneum	Prednisone	MTX + prednisone	20 mg	4	PD	SD	Present (IHC)	Worsening pleuritis/pericarditis and stable MRI head	Alive (52)
5	59/M	Bone, CNS	None	MTX + prednisone	15 mg	4	PD	UNK	ND	Worsening diplopia	UNK
6	49/M	Bone, CNS, lung, CVS, kidney, retroperitoneum	Prednisone Cyt	MTX + prednisone	12.5 mg	12	PR	PD	Present (PCR)	Improved abdominal pain for 6 months, MRI worse after that	UNK
7	59/F	Bone, CNS, kidney, retroperitoneum	Prednisone	MTX + prednisone	20 mg	7	PD	PD	Present (PCR)	New femoral lesions and bone pain	Alive (62)
8	66/F	Orbit, tibia, periaortic area, liver, spleen	None	MTX	UNK	6	PD	UNK	ND	Worsening arthralgia	Dead (67)
9	57/M	Bone, bone marrow, CVS, kidney, retroperitoneum	Prednisone	MTX + infliximab + prednisone	15 mg	3	PD	SD	Present (PCR)	Worsening symptoms but MRI heart stable	Alive (61)
10	49/F	CNS, retroperitoneum	Prednisone	MTX + prednisone	15 mg	24+	PR	SD	Absent (PCR)	Vision improved	Alive (52)
11	34/F	Bone, CNS, skin	None	MTX + infliximab	UNK	25	SD	SD	Present (IHC)	Worsening dizziness and imaging after 25 months	Alive (37)
12	54/M	Bone, CNS, skin, kidney, retroperitoneum	Prednisone	MTX	15 mg	1	PD	PD	Present (urine PCR)^a^	New skin lesions, worsening vasculitis	Alive (57)
13	44/M	Bone, bone marrow, CNS, kidney, retroperitoneum, skin, vas deferens	Tamoxifen Prednisone Azathioprine Cyt	MTX	7.5 mg	163+	PR	SD	ND	Improved vision and paresthesia, stable epidural mass on MRI	Alive (59)

^a^Urine-based circulating tumor DNA test.

*CNS* central nervous system, *CR* complete remission, *CT* computed tomography, *CVS* cardiovascular system, *Cyt* cyclophosphamide, *ECD* Erdheim-Chester disease, *F* female, *IHC* immunohistochemistry, *M* male, *MTX* methotrexate, *MRI* magnetic resonance imaging, *ND* not done, *NE* non-evaluable (due to lack of follow-up visit/imaging), *NGS* next-generation sequencing, *PCR* polymerase chain reaction, *PD* progressive disease, *PET-CT* positron emission tomography–computed tomography, *PR* partial remission, *SD* stable disease, *UNK* unknown

The overall clinical response rate to methotrexate therapy was 23% (0/13 CR and 3/13 PR), with SD in 7% (1/13) and PD in 70% (9/13) patients. Among the three patients who responded to methotrexate therapy, the duration of response was 12, 24, and 163 months, respectively. The latter two patients remained on continuous methotrexate therapy, with ongoing response. Both of them had ECD involving the eyes (subconjunctival and choroidal) and noted improvement in vision with methotrexate. Among the three patients who received methotrexate alone, two had PD and one had PR ongoing after treatment for 163 months, with time to response of 4 months. The overall radiologic response rate was 0%, with SD in 38% (5/13), PD in 46% (6/13), and unknown in 16% (2/13) patients (Table 1). Treatment-related notable adverse effects were seen in one patient who developed CTCAE grade 3 infection (cellulitis requiring hospitalization). Another patient developed grade 3 hyperglycemia that was deemed secondary to the concomitant corticosteroid use. Grade 1 (<3 times upper limit) liver function test abnormalities were noted in three (23%) patients. *BRAF V600E* mutation testing was performed in eight patients, seven of which were positive. Of the seven patients with *BRAF V600E* mutation, six ultimately went on to receive BRAF-inhibitor treatment. At last follow-up, two patients had died, and the causes of death were unknown.

In our series, methotrexate had a low clinical activity. However, the clinical benefit could be sustained, as demonstrated by the two patients who continue to be in remission after 2 and 13 years, respectively. Methotrexate was well tolerated overall, and all treatment discontinuations were due to progressive disease. There were no improvements in radiological manifestations of the disease, even in patients who experienced a clinical response. It is to be noted that both of the patients with ongoing responses did not undergo a PET-CT, which usually is the modality of choice to monitor radiological response^10^. This highlights the underlying chronic fibro-inflammatory nature of the disease that often fails to regress if measured by conventional imaging studies. The two patients who had a treatment response to methotrexate had ocular involvement. The exact reason for activity of methotrexate in ocular ECD is unclear, although there is a previous large study demonstrating its efficacy in other ocular inflammatory diseases as well^11^. In contrast to ocular responses, two other patients who had periorbital disease failed to respond to methotrexate therapy. These findings may indicate its role primarily in ocular ECD.

There are limited existing data on the treatment of ECD with oral methotrexate. One of the first case reports of the use of this agent in ECD involved the use of high-dose intravenous methotrexate for central nervous system disease^12^. This case demonstrated rapid improvement of neurological signs and symptoms in a patient with relapsed ECD who was otherwise declining neurologically. Another case series included one patient with ECD who presented with visual abnormalities and exophthalmos due to periorbital disease that worsened despite therapy with methotrexate^6^. A recently published ECD series included two patients who achieved disease stability with methotrexate, and no objective responses were reported^13^.

The main limitation of our study can be ascribed to it being a single-institution retrospective study that might limit the generalizability of our results. Our study included patients from the past 18 years, and there is heterogeneity in terms of advances in imaging, histopathological techniques of diagnosis, as well as supportive care that cannot be accounted for. In addition, post-therapy response assessment was not uniformly performed in a prospective manner. However, to minimize the chances of errors or bias, the charts were independently reviewed by three investigators.

To our knowledge, this study is the largest series reviewing the efficacy of oral methotrexate in ECD. This highlights the challenges in conducting large ECD studies owing to the rarity of this condition. If no MAP kinase alterations are identified, the role of methotrexate is perhaps limited to second-line or third-line therapies in the presence of established agents like interferon alpha and perhaps cladribine. However, it may be considered a treatment option in primarily ocular ECD. Needless to say, in the right context, it may serve as the treatment of choice depending on patient preference, toxicity profile, and ease of administration. Our study also calls for more studies on other therapeutic targets and novel agents in ECD patients who do not possess MAP-kinase alterations.
